# The efficacy and safety of prokinetic agents in critically ill patients receiving enteral nutrition: a systematic review and meta-analysis of randomized trials

**DOI:** 10.1186/s13054-016-1441-z

**Published:** 2016-08-15

**Authors:** Kim Lewis, Zuhoor Alqahtani, Lauralyn Mcintyre, Saleh Almenawer, Fayez Alshamsi, Andrew Rhodes, Laura Evans, Derek C. Angus, Waleed Alhazzani

**Affiliations:** 1 Department of Medicine, McMaster University, 1280 Main St West, Hamilton, Ontario L8S 4L8 Canada; 2 Department of Clinical Epidemiology and Biostatistics, McMaster University, 1280 Main St West, Hamilton, Ontario L8S 4L8 Canada; 3 Department of Medicine (Critical Care), The Ottawa Hospital Research Institute, University of Ottawa, 1053 Carling Avenue, Rm F202, Ottawa, Ontario K1H 8L6 Canada; 4 Department of Surgery, McMaster University, 1280 Main St West, Hamilton, Ontario L8S 4L8 Canada; 5 Department of Internal Medicine, College of Medicine and Health Sciences, United Arab Emirates University, PO Box 15551, Al-Ain, United Arab Emirates; 6 Department of Intensive Care Medicine, St George’s Hospital, Blackshaw Road, London, SW170QT UK; 7 Department of Medicine, Division of Pulmonary Medicine and Critical Care, New York University, 550 First Avenue, New York City, NY 10016 USA; 8 Department of Critical Care Medicine, University of Pittsburgh School of Medicine, 4200 Fifth Ave, Pittsburgh, Pennsylvania 15260 USA; 9 Department of Medicine, Division of Critical Care, McMaster University, St Joseph’s Healthcare Hamilton, 50 Charlton Avenue, Hamilton, Ontario L8N 4A6 Canada

**Keywords:** Prokinetic agents, Critical illness, Gastroparesis, Enteral nutrition, Systematic review

## Abstract

**Background:**

Intolerance to enteral nutrition is common in critically ill adults, and may result in significant morbidity including ileus, abdominal distension, vomiting and potential aspiration events. Prokinetic agents are prescribed to improve gastric emptying. However, the efficacy and safety of these agents in critically ill patients is not well-defined. Therefore, we conducted a systematic review and meta-analysis to determine the efficacy and safety of prokinetic agents in critically ill patients.

**Methods:**

We searched MEDLINE, EMBASE, and Cochrane Library from inception up to January 2016. Eligible studies included randomized controlled trials (RCTs) of critically ill adults assigned to receive a prokinetic agent or placebo, and that reported relevant clinical outcomes. Two independent reviewers screened potentially eligible articles, selected eligible studies, and abstracted pertinent data. We calculated pooled relative risk (RR) for dichotomous outcomes and mean difference for continuous outcomes, with the corresponding 95 % confidence interval (CI). We assessed risk of bias using Cochrane risk of bias tool, and the quality of evidence using grading of recommendations assessment, development, and evaluation (GRADE) methodology.

**Results:**

Thirteen RCTs (enrolling 1341 patients) met our inclusion criteria. Prokinetic agents significantly reduced feeding intolerance (RR 0.73, 95 % CI 0.55, 0.97; *P* = 0.03; moderate certainty), which translated to 17.3 % (95 % CI 5, 26.8 %) absolute reduction in feeding intolerance. Prokinetics also reduced the risk of developing high gastric residual volumes (RR 0.69; 95 % CI 0.52, 0.91; *P* = 0.009; moderate quality) and increased the success of post-pyloric feeding tube placement (RR 1.60, 95 % CI 1.17, 2.21; *P* = 0.004; moderate quality). There was no significant improvement in the risk of vomiting, diarrhea, intensive care unit (ICU) length of stay or mortality. Prokinetic agents also did not significantly increase the rate of diarrhea.

**Conclusion:**

There is moderate-quality evidence that prokinetic agents reduce feeding intolerance in critically ill patients compared to placebo or no intervention. However, the impact on other clinical outcomes such as pneumonia, mortality, and ICU length of stay is unclear.

**Electronic supplementary material:**

The online version of this article (doi:10.1186/s13054-016-1441-z) contains supplementary material, which is available to authorized users.

## Background

Delayed gastric emptying is common in critically ill patients as a result of many factors including medications (e.g., narcotics, catecholamines), hyperglycemia, renal dysfunction, mechanical ventilation, or the disease process itself [1–5]. When gastric emptying was measured in critically ill patients, 46 % of them had evidence of delayed gastric emptying [6]. Untreated slow gastric emptying has a plethora of clinical consequences such as vomiting, aspiration of gastric contents, pneumonia, and inadequate provision of calories [7–12]. Studies have shown an association between feeding intolerance, prolonged intensive care unit (ICU) stay, and increased risk of death [12, 13]. Although it is possible that this association is a reflection of the underlying severity of illness or a consequence of other unmeasured confounders, feeding intolerance could be playing a causal role. Despite these risks, enteral feeding is preferred to parenteral nutrition as it is associated with fewer septic complications, lower risk of bacterial translocation, and is cheaper [14–21]. There are several therapeutic options that help to overcome feeding intolerance. Enteral nutrition via a small bowel feeding tube may reduce the risk of pneumonia, without compromising nutrition delivery [22]; however, small bowel feeding tubes require technical expertise and are not always available. Prokinetic agents are used for treating non-critically ill patients with gastroparesis [6–8]. The use of these agents in the ICU, although common, is based on unclear evidence.

Metoclopramide, erythromycin and domperidone are the commonest prokinetic agents. Metoclopramide is a selective D2 (dopamine) receptor antagonist that enhances peristalsis in the upper gastrointestinal (GI) tract [23]. Domperidone is another D2 receptor antagonist that increases the amplitude of esophageal motor function and duodenal contractions, and coordinates peristalsis across the pylorus to accelerate gastric emptying [24]. Erythromycin acts locally to enhance the release of motilin from enterochromaffin cells of the duodenum. Motilin causes contraction of the duodenum and gastric antrum [24]. These agents have all been shown to prolong the QT interval, and may cause serious arrhythmias [25].

The most recent systematic review in this area was published more than a decade ago [26]. Since then, multiple randomized trials have been published [27–31]. In addition, cisapride has been withdrawn from the market due to increased risk of arrhythmia and death [32]. Moreover, authors did not focus on clinical outcomes and incorporated studies using acetaminophen absorption as a surrogate marker for gastric motility [33–36]. Our study is an up-to-date systematic review and meta-analysis of randomized trials aiming to determine the efficacy of prokinetic agents use in critically ill adults.

## Methods

### Study selection

Studies were eligible if: (1) the study design was a parallel-group randomized controlled trial (RCT); (2) the population included adult critically ill patients admitted to the ICU who received enteral nutrition; (3) the intervention group received a prokinetic agent, either domperidone, metoclopramide, or erythromycin, regardless of the dose, frequency, and duration; (4) the control group received either no intervention or a placebo; and (5) the outcomes included any of the following: mortality, aspiration, pneumonia, ICU length of stay, vomiting, diarrhea, high gastric residual volume (GRV), feeding intolerance, post-pyloric feeding tube placement, or malignant arrhythmia. We excluded RCTs that compared prokinetic agents, and studies that did not report clinical outcomes.

For our purposes, feeding intolerance was defined as either GRV ≥150 ml, vomiting, or abdominal distention resulting in feeding interruption. For pneumonia outcome we did not mandate meeting a specific definition; there is no universally accepted definition of ventilator-associated pneumonia. Mortality was restricted to mortality in the ICU or hospital mortality. A successful feeding tube insertion was one that was radiologically or endoscopically confirmed to be post-pyloric, in any segment of the duodenum or jejunum.

### Search strategy

We searched MEDLINE, EMBASE, and the Cochrane Library from inception until January 2016. The search strategy is detailed in Additional file 1: Table S1. We screened citations of all potentially eligible articles without language or publication date restrictions. Two reviewers screened titles and abstracts to identify articles for full review, and evaluated the full text of potentially eligible studies. In addition, reviewers screened the reference list of review articles for additional studies. Disagreements between reviewers were resolved by consensus, and if necessary, consultation with a third reviewer.

### Data extraction

Two reviewers independently used a predesigned data abstraction form to extract patients’ demographic data, and data on interventions, outcomes, risk of bias, and other relevant information. Disagreements were resolved by discussion and consensus. We contacted study authors for missing or unclear information.

### Risk of bias

Two reviewers independently assessed trials for risk of bias using the Cochrane risk of bias tool [37]. For each included trial, we judged articles as low, unclear, or high risk of bias for the domains of adequate sequence generation, allocation concealment, blinding of participants and personnel, blinding of outcome assessment, incomplete outcome data, selective outcome reporting, and for other bias. The overall risk of bias for each included trial was categorized as low if the risk of bias is low in all domains, unclear if the risk of bias was unclear in at least one domain and with no high risk of bias domain, or high if the risk of bias was high in at least one domain. We resolved disagreements by discussion and consensus.

### Statistical analysis

We analyzed data using RevMan software (Review Manager, version 5.3. Copenhagen: The Nordic Cochrane Centre, The Cochrane Collaboration, 2014). We used the DerSimonian and Laird random-effects model to pool the weighted effect of estimates across all studies [38]. We estimated study weights using the inverse variance method. We calculated pooled relative risk (RR) for dichotomous outcomes and mean differences (MD) for continuous outcomes, with a corresponding 95 % confidence interval (CI). We inspected funnel plots to assess for publication bias [39].

### Heterogeneity and subgroup analysis

We assessed statistical heterogeneity using the Chi^2^ and I^2^ statistics. We considered Chi^2^ < 0.1 or I^2^ > 50 % as significant heterogeneity [40]. We explored heterogeneity between studies by performing predetermined subgroup analyses to investigate whether certain factors influenced treatment effect. These subgroups included: feeding intolerance and GRV definitions (GRV >250 ml vs GRV >150 ml), class of agents used (metoclopramide vs erythromycin), and subgroup analysis by risk of bias (low risk vs unclear or high risk).

## Results

### Characteristics of included studies

**Fig. 1:**
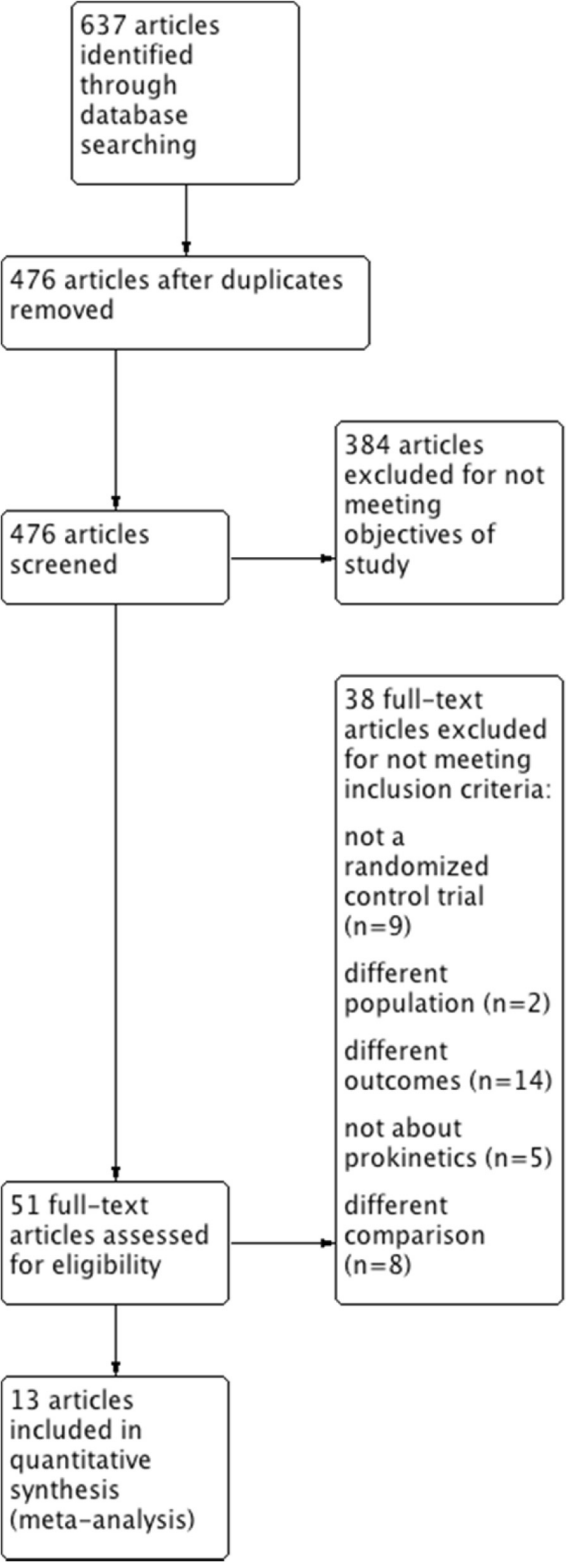
Preferred reporting items for systematic reviews and meta-analyses (PRISMA) flow chart. Description of the study selection process

Our initial search identified a total of 637 citations. After removing duplications 476 publications remained. Of those, 51 underwent a full text review. Another 38 were then excluded for a variety of reasons (Additional file 1: Table S2). Thirteen studies met the inclusion criteria and were included in the quantitative analysis [27–31, 41–48] (Fig. 1). The details of eligible trails are presented in Table 1. A total of 1341 patients were enrolled in 13 RCTs that included a variety of critically ill patients with medical, surgical, and neurosurgical conditions. Eight trials used intravenous (IV) metoclopramide at different frequencies. Seven RCTs used erythromycin at a range of doses and administration schedules. Two studies used domperidone, one of which did not indicate the dose, route of administration, or frequency. Other details of eligible studies are presented in Table 1.

**Table 1:** Characteristics of studies

Author	Population	Feeding intolerance at baseline	Intervention groups	Outcomes	Definition of feeding intolerance	Definition of nosocomial pneumonia	Funding
Whatley 1984 USA (n = 10)	Critically ill patients who failed post-pyloric tube insertion Mean age: 46.0 years, 80.0 % male Mean APACHE II score: not reported	No	Metoclopramide 20 mg IV single dose vs no intervention	1) Successful post-pyloric feeding tube insertion	N/A	N/A	NR
Heiselman 1995 USA (n = 105)	Critically ill patients who required enteral nutrition Mean age: NR % male: NR Mean APACHE II: NR	No	Metoclopramide 10 mg IV vs no medication	1) Successful post-pyloric feeding tube insertion	N/A	N/A	NR
Kalliafas 1996 USA (n = 57)	Critically ill patients who required enteral nutrition Mean age: 57.4 years, 52.6 % male Mean APACHE II score 14.9	No	Erythromycin 200 mg IV vs placebo	1) Successful post-pyloric feeding tube insertion	N/A	N/A	NR
Paz 1996 USA (n = 83)	Critically ill patients who required enteral nutrition Mean age: 60.8 years, 53.0 % male Mean APACHE II score: NR	No	Erythromycin 200 mg IV single dose vs metoclopramide 10 mg IV single dose vs placebo	1) Successful postpyloric feeding tube insertion	N/A	N/A	Industry
Chapman 2000 Australia (n = 20)	Critically ill, mechanically ventilated patients who failed enteral feeding Mean age: 46.3 years, 80 % male Mean APACHE II score:16.2	Yes	Erythromycin 200 mg IV single dose vs placebo	1) Mortality 2) Feeding intolerance	Gastric residual volume greater than or equal to 250 ml	N/A	NR
Yavagal 2000 India (n = 305)	Critically ill patients who required a nasogastric tube for more than 24 hours Mean age: 36.5 years, 62.0 % male Mean APACHE II score: 17.7	No	Metoclopramide 10 mg IV q8h vs placebo	1) Mortality 2) Nosocomial pneumonia	N/A	1) New infiltrate of chest radiograph 2) A positive tracheal or sputum culture 3) Axillary temperature greater than 38 °C 4) Leukocytosis (white cell count greater than 12,000/ml) or Leukopenia (white cell count less than 3000/ml)	NR
Pinilla 2001 Canada (n = 80)	Critically ill patients who required enteral nutrition for 3 or more days Mean age: 52.9 years, 55.0 % male Mean APACHE II score: NR	No	Metoclopramide, cisapride or domperidone (no dose/route/frequency provided) vs no intervention	1) Gastrointestinal intolerance 2) Vomiting 3) Gastric residual volumes 4) Diarrhea	1) Witnessed vomiting 2) Diarrhea (3 or more liquid stools in a 24-hour period) 3) Gastric residual volume greater than 150 ml for the control group or greater than 250 mL for the treatment group	N/A	NR
Berne 2002 USA (n = 68)	Critically ill trauma patients who had a gastric residual volume greater than 150 ml in the 1^st^ 48 hours of feeding Mean age: 37.1 years, 84.7 % male Mean ISS: 24.2	Yes	Erythromycin 250 mg IVq6h vs placebo	1) Nosocomial pneumonia 2) Feeding intolerance 3) ICU length of stay 4) Mortality 5) Infectious complications	Gastric residual volumes greater than 150 ml	1) Fever greater than 38.6 °C, 2) Leukocytosis (white blood cells greater than 10,000 cell/L) 3) Purulent sputum 4) New infiltrate on chest radiograph 5) Sputum sample showing moderate or many white blood cells and a positive culture	NR
Reignier 2002 France (n = 40)	Critically ill patients receiving mechanical ventilation and early nasogastric feeding Mean age: 68.0 years, 50.0 % male Mean APACHE II score: NR	No	Erythromycin 250 mg IV q6h × 5 days vs D5W 50 ml IV q6h × 5 days	1) Mortality 2) Gastric intolerance 3) Vomiting	1) Vomiting 2) Gastric residual volume greater than 250 ml	N/A	NR
Griffith 2003 USA (n = 36)	Critically ill patients requiring enteral nutrition and exhibiting one or more of: evidence of delayed gastric emptying with repeatedly high gastric aspirates, history of pulmonary aspiration of tube feeds, clinical high risk of aspiration, head-of-the-bed elevation not possible, or severe acute pulmonary disease Mean age: 57.2 years, 69.4 % male Mean APACHE II score: NR	Yes	Erythromycin 500 mg IV single dose vs placebo	1) Successful post-pyloric feeding tube insertion	N/A	N/A	Academic
Nursal 2007 Turkey (n = 19)	Critically ill patients Mean age: 43.4 years, 84.2 % male Mean APACHE II: 12.9		Metoclopramide 10 mg IV TID × 5 days vs normal saline TID × 5 days	1) Mortality 2) Aspirations 3) Feeding intolerance 4) ICU length of stay 5) Vomiting 6) Ileus 7) Diarrhea 8) Gastric residual volume 9) Extrapyramidal movement	1) Gastric residue volume greater than twice the current hourly infusion rate, or if it was more than 150 ml 2) Abdominal distention, vomiting, or diarrhea	N/A	NR
Nassaj 2010 (n = 220)	Critically ill patients who required a nasogastric tube for more than 24 hours Mean age: 44.0 years, 65.5 % male Mean APACHE II score: 14.9	No	Metoclopramide 10 mg PO q8h × 5 days vs no intervention	1) Nosocomial pneumonia 2) Mortality	N/A	1) Axillary temperature greater than 37.5 °C 2) Leukocytosis (white blood cells greater than 11, 000 cell/L) 3) Increase in tracheal secretions (>0.4 cm^3^/hour) 4) New infiltrate on the chest radiograph or progression of an existing infiltrate	NR
Hu 2015 China (n = 298)	Critically ill patients who required enteral nutrition for more than 3 days Mean age: 62.9 years, 66.1 % male Mean APACHE II score: 21.1	No	Metoclopramide 20 mg IV (single dose) vs domperidone 20 mg QID vs no intervention	1) Successful post-pyloric feeding tube insertion	N/A	N/A	Academic

*APACHE* acute physiology, age and chronic health evaluation, *ICU* intensive care unit, *IV* intravenous, *N/A* not applicable, *NR* not reported, *PO* per os, *QID* four times a day, *TID* three times a day

### Risk of bias

**Fig. 2:**
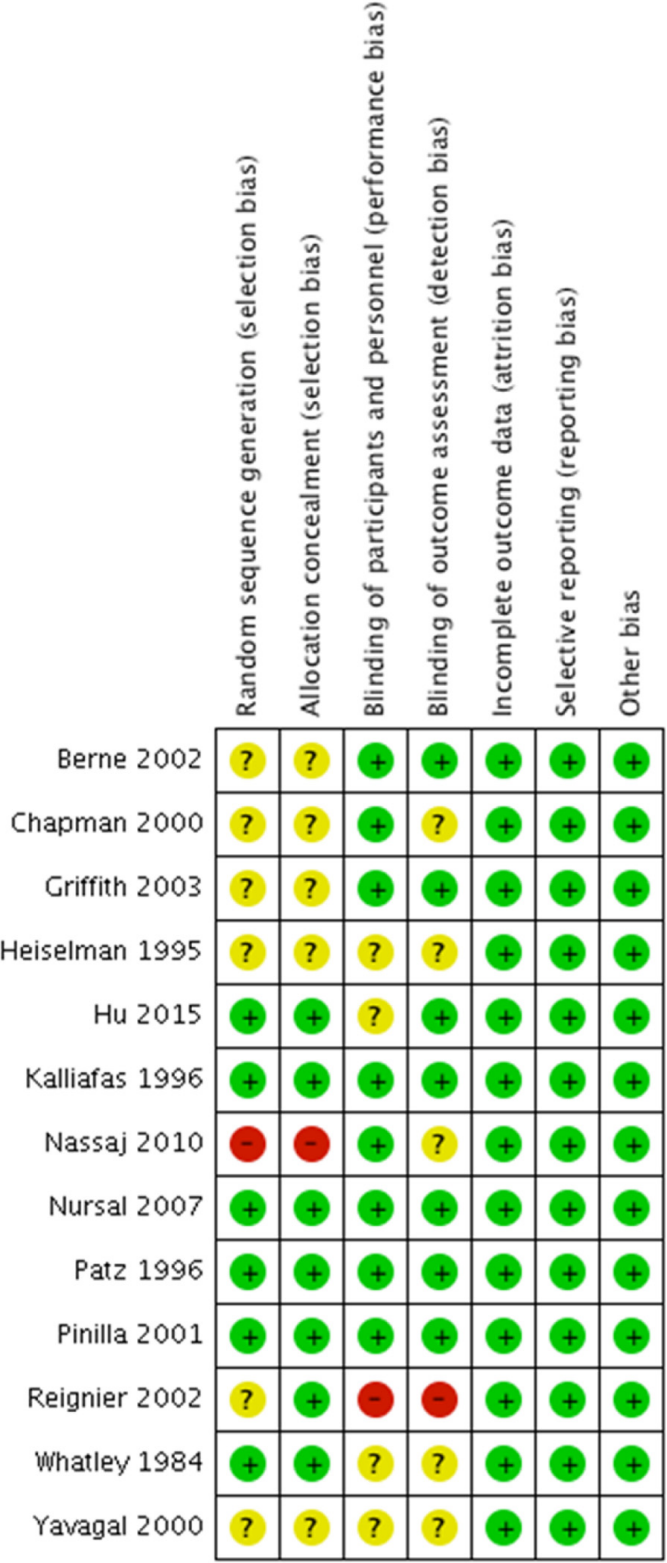
Risk of bias assessment. Summary of risk of bias in individual randomized controlled trials in this systematic review; *green circles* low risk of bias, *yellow circles* unclear risk of bias, *red circles* indicate high risk of bias

Using the Cochrane risk-of-bias tool, we judged two studies to be at high risk of bias due to inappropriate randomization and blinding methods [30, 48], five studies were at low risk of bias [29, 31, 43–45], and we were not able to comprehensively assess risk of bias in six studies due to lack of information [27, 28, 41, 42, 46, 47]. The details of risk of bias assessment are presented in Fig. 2.

### Publication bias

We inspected funnel plots for each outcome for asymmetry; however, we included fewer than 10 RCTs in each outcome, therefore, the test is underpowered to reliably detect evidence of publication bias (Additional file 1: Figures S1 and S2).

### Main outcomes

**Fig. 3:**
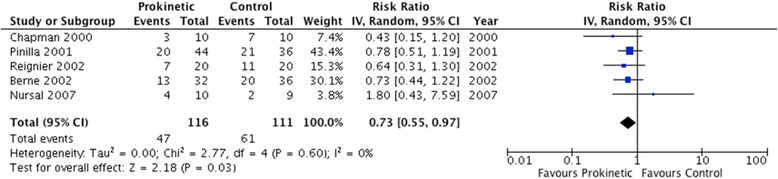
Feeding intolerance outcome. Forest plot includes pooled estimates for randomized controlled trials comparing prokinetic agents to placebo or no intervention for feeding intolerance outcome. *IV* inverse variance, *CI* confidence interval

**Fig. 4:**
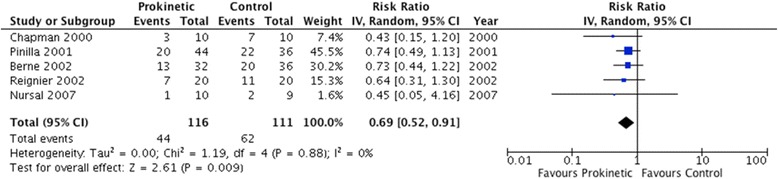
High gastric residual volume (>250 ml). Forest plot includes pooled estimates for randomized controlled trials comparing prokinetic agents to placebo or no intervention for high gastric residual volume outcome. *IV* inverse variance, *CI* confidence interval

**Fig. 5:**
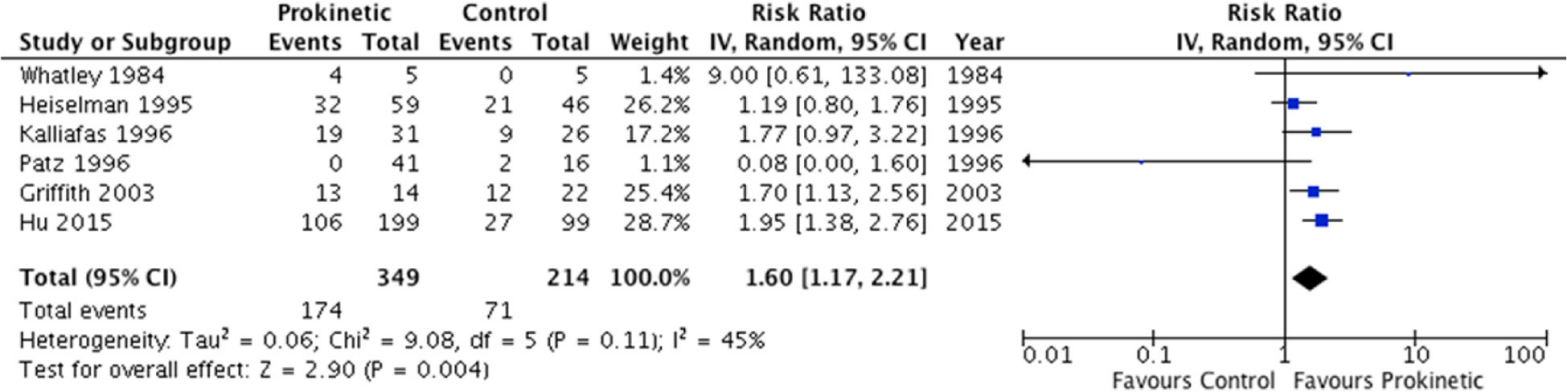
Successful post-pyloric feeding tube placement. Forest plot includes pooled estimates for randomized controlled trials comparing prokinetic agents to placebo or no intervention for successful insertion of post-pyloric tube outcome. *IV* inverse variance, *CI* confidence interval

**Fig. 6:**
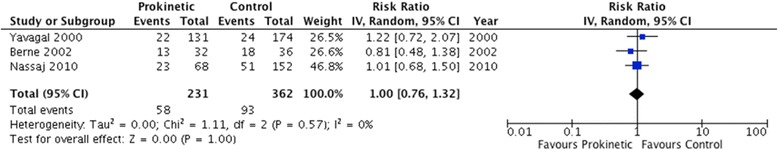
ICU-acquired pneumonia outcome. Forest plot includes pooled estimates for randomized controlled trials comparing prokinetic agents to placebo or no intervention for ICU-acquired pneumonia outcome. *IV* inverse variance, *CI* confidence interval

The use of prokinetic agents significantly reduced feeding intolerance, as assessed by five studies enrolling 227 patients (RR 0.73, 95 % CI 0.55, 0.97; *P* = 0.03; *I*^2^ = 0 %) (Fig. 3). The number needed to treat (NNT) was 12 (95 % CI 7, 111). In addition, prokinetics significantly reduced the risk of developing high GRV (RR 0.69, 95 % CI 0.52, 0.91; *P* = 0.009; *I*^2^ = 0 %) (Fig. 4). Prokinetics significantly increased the rate of successful post-pyloric feeding tube placement (RR 1.60, 95 % CI 1.17, 2.21; *P* = 0.004; *I*^2^ = 46 %) (Fig. 5). Compared to placebo, prokinetic agents did not prevent the development of pneumonia (RR 1.00, 95 % CI 0.76, 1.32; *P* = 0.57; *I*^2^ = 0 %) (Fig. 6), nor reduced the risk of death (RR 0.97, 95 % CI 0.81, 1.16; *P* = 0.72; *I*^2^ = 0 %) (Additional file 1: Figure S3) or length of ICU stay (RR 1.24, 95 % CI −5.21, 7.68, *P* = 0.43; *I*^2^ = 0 %) (Additional file 1: Figure S4). However, there was a non-significant reduction in vomiting with the use of prokinetic agents (RR 0.74, 95 % CI 0.49, 1.12, *P* = 0.15; *I*^2^ = 0 %) (Additional file 1: Figure S5). Finally, prokinetics did not significantly increase the risk of diarrhea compared to placebo (RR 1.82, 95 % CI 0.67, 4.91; *P* = 0.24; *I*^2^ = 0 %) (Additional file 1: Figure S6).

### Subgroup analysis

Despite the absence of statistical heterogeneity, we performed subgroup analyses to determine if there are important subgroup differences. Subgroup analysis by threshold of GRV (>150 ml vs >250 ml) was not significant (Additional file 1: Figure S7). Prokinetic agents did not prevent feeding intolerance in patients without gastroparesis (RR 0.62, 95 % CI 0.31, 1.22, *P* = 0.16), but did reduce feeding intolerance in those with pre-existing gastroparesis (RR 0.70, 95 % CI 0.52, 0.96; *P* = 0.03), however, the interaction test did not reach statistical significance (Additional file 1: Figure S8). Although the treatment effect on feeding intolerance was larger in trials with high risk of bias (RR 0.65, 95 % CI 0.44, 0.96; *P* = 0.03) the interaction test was not significant (Additional file 1: Figure S9). Similarly, there was no significant subgroup difference in any outcomes by drug class, except for erythromycin, which improved feeding intolerance (Additional file 1: Figure S10). We present the results of our subgroup analyses in Additional file 1: Table S3.

### Quality of evidence

The quality of evidence was moderate for feeding intolerance, high GRV, and success in post-pyloric tube placement outcomes. While the quality of evidence was low for other outcomes. The details of risk of bias and quality assessment are outlined in Additional file 1: Table S4 and S5, respectively.

## Discussion

In this systematic review, we included 13 RCTs (1341 patients) in the final analysis. Our results show that the use of prokinetic agents in patients receiving enteral feeding in the ICU improve both feeding intolerance and high GRVs. Furthermore, prokinetic agents were found to increase the success of inserting post-pyloric feeding tubes. Prokinetics did not significantly reduce the risk of pneumonia, mortality, length of stay in the ICU, or vomiting, nor did it increase the risk of diarrhea.

Many studies used acetaminophen absorption as a surrogate for gastric motility [33, 35, 49–53] and were included in prior systematic reviews [26]. Acetaminophen is not absorbed by the stomach but is rapidly absorbed in the intestine [54]. As a result of rapid absorption, measurements such as time to peak acetaminophen concentration and maximum plasma concentration are used to assess gastric motility [54]. Acetaminophen is a convenient test to use in critically ill patients as it simply requires serial blood work. Although acetaminophen absorption has been correlated with scintigraphy assessments [54], the accuracy of the test is questioned. Studies show no significant correlation between the half-time of gastric emptying and peak acetaminophen levels, and therefore it may not correspond clinically [55, 56]. For those reasons, we chose not to use the acetaminophen absorption as a surrogate of feeding intolerance and focused on clinical outcomes.

As previously described, prokinetics are not without potential complications. In the wake of cisapride being withdrawn from the market due to risk of malignant arrhythmia, intravenous erythromycin has been avoided due to its potential to cause serious ventricular arrhythmia [57, 58]. However, arrhythmia occurred at a dose of 3 grams per day, which far exceeds that used for the promotility properties of the drug [27, 28, 41, 43, 48]. Although arrhythmia was not explicitly reported in the RCTs used in this meta-analysis, mortality did not significantly differ between metoclopramide and erythromycin as demonstrated by this systematic review. In addition to cardiac complications, there is also concern about microbial resistance to antibiotics [26]. Berne et al. specifically looked at infectious complications, and found none [27]. Lastly, tachyphylaxis can develop with erythromycin use. This was observed in an RCT showing loss of erythromycin effect after 48 hours of treatment [27]. In another RCT, there was reduction in GRVs at 12 hours, but not beyond [41]. Likewise, another study found no difference in GRVs between the erythromycin and placebo groups at day 5 of the trial [48].

Domperidone is another prokinetic that can be considered and is commonly used in the outpatient setting to treat gastroparesis. However, domperidone is associated with risk of QT interval prolongation [23]. A case–control study of 1608 cases of cardiac arrhythmia and death found there was an increased risk with domperidone use in the outpatient setting, particularly with doses exceeding 30 mg per day (odds ratio (OR) 11.4, 95 % CI 1.99, 65.2) [59]. Only two RCTs in our review used domperidone [29, 45]. In one study physicians were allowed to use erythromycin, domperidone or metoclopramide as their prokinetic to treat feeding intolerance and only one patient received domperidone [29]. Therefore, we cannot make firm conclusions about the efficacy of domperidone. However, domperidone did seem to be as effective as metoclopramide for aiding the insertion of a post-pyloric feeding tube [29].

The Canadian Critical Care Clinical Practice guidelines recommend metoclopramide as the first-line prokinetic agent in the ICU [60]. Several studies suggest that erythromycin may be more effective than metoclopramide [52, 61]. Our subgroup analysis was underpowered to detect any meaningful difference between different agents. The recent American Society of Parenteral and Enteral Nutrition (ASPEN) guidelines suggest using either metoclopramide or erythromycin in patients at high risk of aspiration [62]. Both agents are associated with tachyphylaxis [61, 63]. However, due to the risk of complications of erythromycin, metoclopramide was proposed as the first-line treatment. Moreover, we believe that prokinetics should not be used prophylactically, but only be used to treat patients with feeding intolerance, this is supported by our subgroup analysis demonstrating larger effect in this population.

Prior meta-analysis examined the effects of prokinetics; however, our study is the first to report on clinical outcomes [26]. We conducted a comprehensive search and included more RCTs, therefore, the results are more precise and generalizable. Two independent reviewers performed screening, data abstraction and risk of bias assessment. In addition, we adhered to the PRISMA guidelines (Additional file 1: Table S6) [64]. Finally, we used GRADE methodology to assess the quality of evidence.

Despite the robust results, there are some limitations. Important clinical outcomes (i.e., pneumonia, diarrhea, and vomiting) were not consistently reported in all studies, which resulted in imprecise estimates. Moreover, cardiac and infectious side effects were rarely reported and many RCTs had a short follow-up time, therefore, we could not generate meaningful estimates to help practitioners. In addition, there was inconsistency in the definition of feeding intolerance, and in some studies the definition of feeding intolerance was not clear. Also, the type, dose and frequency of prokinetic agents varied significantly across studies. Last, we were unable to do subgroup analysis on the different ICU patient populations and to discern the outcomes in patients with vs without sepsis. A large RCT is needed to examine any subgroup effect, and to determine the impact on other clinical outcomes, including potential side effects. However, the current evidence supports the use of prokinetic agents to treat feeding intolerance in critically ill patients, in the absence of contraindication to these agents.

## Conclusion

Moderate-quality evidence showed that prokinetic agents are effective in improving feeding intolerance in critically ill patients and in facilitating post-pyloric feeding tube placement, and low-quality evidence failed to demonstrate a significant reduction in pneumonia, vomiting, or mortality. There was also no significant increase in the rates of diarrhea, and no significant arrhythmia was reported in eligible studies. Future RCTs are needed to determine the most effective agent and the impact other important outcomes.

## Abbreviations

ASPEN, American Society of Parenteral and Enteral Nutrition; CI, confidence interval; GI, gastrointestinal; GRADE, grading of recommendations assessment, development, and evaluation; GRV, gastric residual volume; GV, gastric residual volume; ICU, intensive care unit; IV, intravenous; MD, mean difference; NNT, number needed to treat; PRISMA, preferred reporting items for systematic reviews and meta-analyses; RCT, randomized controlled trial; RR, relative risk

## Additional file

Additional file 1: Table S1. Search Strategy. Table S2 Excluded Studies. Table S3 Subgroup Analysis. Table S4 Risk of Bias. Table S5 Quality of Evidence. Table S6 PRISMA checklist. Figure S1 Funnel plot (feeding intolerance outcome). Figure S2p. Funnel plot (high GRV). Figure S3 Mortality outcome. Figure S4 ICU length of stay outcome. Figure S5 Vomiting outcome. Figure S6 Diarrhea outcome. Figure S7 Subgroup analysis by GRV threshold. Figure S8 Subgroup analysis by indication of treatment. Figure S9 Subgroup analysis by risk of bias. Figure S10 subgroup analysis by drug class. (DOCX 2164 kb)
