# Supplementary material for: The efficacy and safety of prokinetic agents in critically ill patients receiving enteral nutrition: a systematic review and meta-analysis of randomized trials
Source: Crit Care. 2016 Aug 15;20:259. doi: 10.1186/s13054-016-1441-z (PMC4986344; doi:10.1186/s13054-016-1441-z)
Supplement: Additional file 1: Table S1. — Search Strategy. Table S2 Excluded Studies. Table S3 Subgroup Analysis. Table S4 Risk of Bias. Table S5 Quality of Evidence. Table S6 PRISMA checklist. Figure S1 Funnel plot (feeding intolerance outcome). Figure S2p. Funnel plot (high GRV). Figure S3 Mortality outcome. Figure S4 ICU length of stay outcome. Figure S5 Vomiting outcome. Figure S6 Diarrhea outcome. Figure S7 Subgroup analysis by GRV threshold. Figure S8 Subgroup analysis by indication of treatment. Figure S9 Subgroup analysis by risk of bias. Figure S10 subgroup analysis by drug class. (DOCX 2164 kb) [file 13054_2016_1441_MOESM1_ESM.docx]

**The Efficacy and Safety of Prokinetic Agents in Critically Ill Patients Receiving Enteral Nutrition: A Systematic Review and Meta-analysis of Randomized Trials**

Kim Lewis^1^, Zuhoor Alqahtani^2^, Lauralyn Mcintyre^3^, Saleh Almenawer^2,4^, Fayez Alshamsi^5^, Andrew Rhodes^6^, Laura Evans^7^, Derek C. Angus^8^, Waleed Alhazzani^1,2^

^1^ Department of Medicine, McMaster University, Hamilton, Canada

^2^ Department of Clinical Epidemiology and Biostatistics, McMaster University, Hamilton, Canada

^3^ Department of Medicine (Critical Care), The Ottawa Hospital Research Institute, University of Ottawa, Ottawa, ON, Canada

^4^ Department of Surgery, McMaster University, Hamilton, Canada

^5^ Department of Critical Care, Al-Ain University, Al-Ain, UAE

^6^ Department of Intensive Care Medicine, St George’s Hospital, London, UK

^7^ Department of Medicine, Division of Pulmonary Medicine and Critical Care, New York University, New York, USA

^8^ Department of Critical Care Medicine, University of Pittsburgh School of Medicine, Pittsburgh, PA, USA

**Correspondence**

Waleed Alhazzani MD, MSc, FRCPC

McMaster University, Department of Medicine, Division of Critical Care

St Joseph’s Healthcare Hamilton

50 Charlton Avenue, Postal Code L8N 4A6, Hamilton, Ontario, Canada

Tel: +1905-522-1155 ext 32800

Fax: +1905-521-6068

Email: [alhazzaw@mcmaster.ca](mailto:alhazzaw@mcmaster.ca)

**Table of Contents**

**Table 1.** Search Strategy

**Table 2.** Excluded Studies

**Table 3.** Subgroup Analysis

**Table 4.** Risk of Bias

**Table 5:** Quality of Evidence

**Table 6.** PRISMA Checklist

**Figure 1.** Funnel Plot (Feeding Intolerance Outcome)

**Figure 2.** Funnel Plot (High GRV)

**Figure 3.** Mortality Outcome

**Figure 4.** ICU Length of Stay Outcome

**Figure 5.** Vomiting Outcome

**Figure 6.** Diarrhea Outcome

**Figure 7.** Subgroup Analysis by GRV Threshold

**Figure 8.** Subgroup Analysis by Indication of Treatment

**Figure 9.** Subgroup Analysis by Risk of Bias

**Figure 10.** Subgroup Analysis by Drug Class

**Table 1. Search Strategy**

Embase <1974 to 2016 January>, Ovid MEDLINE(R) In-Process & Other Non-Indexed Citations, Ovid MEDLINE(R) Daily and Ovid MEDLINE(R) <1946 to Present>

--------------------------------------------------------------------------------

1 Critical Illness/ (42949)

2 Critical Care/ or Intensive Care/ (142348)

3 Intensive Care Units/ (132512)

4 (critical care or intensive care).tw,hw. (405301)

5 (critical$ adj3 ill$).tw,hw. (106054)

6 exp Sepsis/ (292653)

7 (sepsis or septic patient$ or septicem$ or septicaem$ or septic shock or Bacteremia or endotoxemi$).tw. (278500)

8 1 or 2 or 3 or 4 or 5 or 6 or 7 (798147)

9 Enteral Nutrition/ (38109)

10 ((alimentary canal or gastrointestinal or enteral$ or enteric or oral$ or sip or gastric or tube$) adj3 (nutrition$ or feed$ or diet$ or fed)).tw,hw. (82330)

11 enteral$ administration.tw. (908)

12 ((post-pylor$ postpylor$ or nasogastric or gastrostomy or jejunostomy or gastric or orogastric or nasoenteric or nasojejunal or feed$) adj3 tube$).tw,hw. (39162)

13 9 or 10 or 11 or 12 (101937)

14 8 and 13 (15906)

15 exp Erythromycin/ (88222)

16 (erythromycin adj5 (gastric or gastrointestin$)).tw,hw. (928)

17 (Metoclopramide or domperidone).tw. (16281)

18 prokinetic.mp. (6546)

19 motility agent$.mp. (200)

20 motilin receptor.mp. or exp Motilin/ (3555)

21 motilin receptor agonist.mp. (184)

22 15 or 16 or 17 or 18 or 19 or 20 or 21 (112081)

23 14 and 22 (574)

**Table 2. Excluded Studies**

| **Article Number** | **Full reference** | **Reason for Exclusion** |
| --- | --- | --- |
| 1 | Tu C, Tsai, c, Huang T, Sheng S, Liu T.Postoperative ileus in the elderly.  International Journal of Gerontology. 2014;8:1-5. | Different population. The patients were non-critically ill. |
| 2 | Kesey J, Dissanaike S. A protocol of early aggressive acceleration of tube feeding increases ileus without perceptible benefit in severely burned patients. Journal of Burn Care and Research. 2013;34:515-520. | Non-randomized study |
| 3 | Deane A, Summers M, Zaknic A, Wong G, Di Bartolomeo A, Sim J, Chapman M, Fraser R, Horowitz M. The effects of erythromycin on nutrient absorption and small intestinal transit in the critically ill. Critical Care Medicine. 2011;39:11. | Outcomes not reported |
| 4 | Schorghuber M, Reintam Blaser A, Starkopf J, Tatzl E, Fruhwald S. Diagnosis and management of gastrointestinal (GI)-motility disorders-results of a survey among austrian and estonian intensive care physicians (ICP).  Intensive Care Medicine. 2012;38:S145. | Non-randomized study |
| 5 | Mechanick J, Berger M. Convergent evidence and opinion on intensive metabolic support.  Current Opinion in Clinical Nutrition and Metabolic Care. 2012;15:144-146. | Review article |
| 6 | Reignier J, Mercier E, Desachy A, et al. Impact of not monitoring gastric volume in mechanically ventilated patients receiving early enteral feeding: A multicenter randomized trial.  Intensive Care Medicine. 2011;37:S295 | Different intervention |
| 7 | Joly F, Amiot A, Messing B. Nutritional Support in the Severely Compromised Motility Patient: When and How? Gastroenterology Clinics of North America. 2011;40:846-851. | Different population |
| 8 | Nguyen N, Grgurinovich N, Bryant L, et al. Plasma erythromycin concentrations predict feeding outcomes in critically ill patients with feed intolerance. Critical Care Medicine. 2011;39:868-871. | Non-randomized study  Outcomes not reported |
| 9 | Ogungbenro K, Vasist L, Maclaren R, Dukes G, Young M, Aarons L. A semi-mechanistic gastric emptying model for the population pharmacokinetic analysis of orally administered acetaminophen in critically ill patients. Pharmaceutical Research. 2011;28:394-404. | Outcomes not reported |
| 10 | Reignier J, Vinatier I, Martin-Lefvre L, Clementi E, Fiancette M. Enteral feeding in patients treated with mechanical ventilation in the prone position. <Nutrition enterale et ventilation mecanique en decubitus ventral.>  Reanimation. 2010;19:454-459. | Non-randomized trial |
| 11 | Van Der Spoel J, Oudemans-Van Straaten H. Editorial: Ubi poop, ibi evacua? Critical Care Medicine. 2010;38:2064-2065. | Editorial |
| 12 | Lu N, Zheng R, Lin H, et al. Study of erythromycin and metoclopramide in treatment of feeding intolerance of critically ill patients in intensive care unit. Chinese Critical Care Medicine. 2010;22:36-39. | Different control |
| 13 | Reignier J, Dimet J, Martin-Lefevre L, et al. Before-after study of a standardized ICU protocol for early enteral feeding in patients turned in the prone position. Clinical Nutrition. 2010;29: 210-216. | Non-randomized study |
| 14 | Nguyen N, Fraser R, Bryant L, et al. Effects of metoclopramide and erythromycin prokinetic treatment on plalphaSMA motilin concentrations in critically ill patients. Journal of Gastroenterology and Hepatology. 2010;25: A25. | Outcomes not reported |
| 15 | Nguyen N, Fraser R, Bryant L, et al. Plasma motilin responses to prokinetic therapy in feed intolerant critically Ill patients. Gastroenterology. 2010;138:S406. | Outcomes not reported |
| 16 | Elliot S, Beardow Z, Ahmed S, Mallick A. Electromagnetic sensor guided nasojejunal tube placement in critically ill patients. Intensive Care Medicine. 2010;36:S374. | Different intervention |
| 17 | Gopal V., Hutchinson S. Achieving nutritional targets in the ICU. Critical Care. 2010;14:S185. | Outcomes not reported |
| 18 | Thomson A. A window into the belly: Lessons from a pioneering surgeon. Journal of Surgery. 2009;79:7-8. | Different intervention |
| 19 | Nguyen N, Fraser R, Bryant L, et al. Plasma erythromycin predicts feeding outcomes in critically ill patients with feed intolerance.  Journal of Gastroenterology and Hepatology. 2009;24:A327-A328. | Outcomes not reported |
| 20 | MacLaren R, Kier T, Fish D, Wischmeyer P. Erythromycin vs metoclopramide for facilitating gastric emptying and tolerance to intragastric nutrition in critically ill patients.  Journal of Parenteral and Enteral Nutrition. 2008;32: 412-419. | Different control |
| 21 | Landzinski J, Kiser T, Fish D, Wischmeyer P, MacLaren R. Gastric motility function in critically ill patients tolerant vs intolerant to gastric nutrition. Journal of Parenteral and Enteral Nutrition. 2008;32: 45-50. | Outcomes not reported |
| 22 | Nguyen N, Chapman M, Fraser R, Bryant L, Burgstad C, Holloway R. Prokinetic therapy for feed intolerance in critical illness: One drug or two? Critical Care Medicine. 2007;35: 2561-2567. | Different control |
| 23 | Nguyen N, Mangoni A, Fraser R, et al. Prokinetic therapy with erythromycin has no significant impact on blood pressure and heart rate in critically ill patients. British Journal of Clinical Pharmacology. 2007;63: 498-500. | Outcomes not reported |
| 24 | Nguyen N, Chapman M, Fraser R, Bryant L, Holloway R. Erythromycin is more effective than metoclopramide in the treatment of feed intolerance in critical illness. Critical Care Medicine. 2007;35:483-489. | Different control |
| 25 | Binnekade J, Tepaske R, Bruynzeel P, Mathus-Vliegen E, de Hann R. Daily enteral feeding practice on the ICU: attainment of goals and interfering factors. Critical care. 2005; 9:R218-225. | Non-relevant study |
| 26 | Ritz M, Chapman M, Fraser R, et al. Erythromycin dose of 70 mg accelerates gastric emptying as effectively as 200 mg in the critically ill. Intensive Care Medicine. 2005;31:949-954. | Different control |
| 27 | Sustic A, Zelic M, Protic A, Zupan Z, Simic O, Desa K. Metoclopramide improves gastric but not gallbladder emptying in cardiac surgery patients with early intragastric enteral feeding: Randomized controlled trial. Croatian Medical Journal. 2005;46:239-244. | Outcomes not reported |
| 28 | MacLaren R, Patrick W, Hall R, Rocker G, Whelan G, Lima J. Comparison of cisapride and metoclopramide for facilitating gastric emptying and improving tolerance to intragastric enteral nutrition in critically ill, mechanically ventilated adults. Clinical Therapeutics. 2001;23:1855-1866. | Different control |
| 29 | Liu K, Atten M, Skipper A, Kumar S, Olson D, Schwenk W. Nutrition in the pediatric population, older adults, and obese patients: Part IV. Enteral nutrition support. Disease-a-Month. 2002;48:745-790. | Review article |
| 30 | Boivin M, Levy H. Gastric feeding with erythromycin is equivalent to transpyloric feeding in the critically ill. Critical Care Medicine. 2001;29:1916-1919. | Different control |
| 31 | Bastian L, Weimann A. Practical aspects of early enteral nutrition. <Praktische aspekte der fruhen enteralen ernahrung.> Journal fur Anasthesie und Intensivbehandlung. 2001;8:221-226. | Review article |
| 32 | MacLaren R, Kuhl D, Gervasio J, et al. Sequential single doses of cisapride, erythromycin, and metoclopramide in critically ill patients intolerant to enteral nutrition: A randomized, placebo-controlled, crossover study. Critical Care Medicine. 2000;28:438-444. | Outcomes not reported |
| 33 | Engle K, Hannawa T. Techniques for administering oral medications to critical care patients receiving continuous enteral nutrition.  American Journal of Health-System Pharmacy. 1999;56:1441-1444. | Non relevant study |
| 34 | Jooste C, Mustoe J, Collee G. Metoclopramide improves gastric motility in critically ill patients. Intensive Care Medicine. 1999;25:464-468. | Outcomes not reported |
| 35 | Wilmer A, Dits H, Malbrain M, Frans E, Tack J, Frost P. Gastric emptying in the critically ill - the way forward (multiple letters). Intensive Care Medicine. 1997;23:928-929. | Letter |
| 36 | Deane A, Wong G, Horowitz M, et al. Randomized double-blind crossover study to determine the effects of erythromycin on small intestinal nutrient absorption and transit in the critically ill. American Journal of Clinical Nutrition. 2012;95:1396-1402. | Outcomes not reported |
| 37 | Nguyen N, Ching K, Fraser R, Chapman M, Holloway R. Risk of Clostridium difficile diarrhoea in critically ill patients treated with erythromycin-based prokinetic therapy for feed intolerance. Intensive Care Medicine. 2008;34:169-173. | Non-randomized study |
| 38 | Weekes J. Erythromycin to facilitate placement of naso-duodenal feeding tubes. Anaesthesia & Intensive Care. 1994;22:318. | Letter |

In this table we present the list of excluded studies after full-text review, with reasons of exclusion.

**Table 3. Subgroup Analysis**

| **Subgroup** | **Subtotal; n** | **Relative Risk**  **(95% CI)** | **P value** | **I^2^ (heterogeneity between groups)** |
| --- | --- | --- | --- | --- |
| Methodologic quality of studies  Low risk of bias    High or unclear risk of bias | 99  128 | 0.88 (0.49, 1.58)  0.65 (0.44, 0.96) | 0.68  0.03 | 0% |
| GRV definition  150ml  250ml | 87  60 | 0.71 (0.43, 1.17)  0.56 (0.31, 1.01) | 0.07  0.06 | 0% |
| Indication  Prophylactic  Pre-existing intolerance | 59  168 | 0.62 (0.31, 1.22)  0.69 (0.52, 0.91) | 0.16  0.03 | 0% |
| Agent (Mortality)  Metoclopramide  Erythromycin | 544  128 | 0.95 (0.74, 1.22)  0.83 (0.41, 1.70) | 0.66  0.62 | 0% |
| Agent (Vomiting)  Metoclopramide  Erythromycin | 19  40 | 0.45 (0.05, 4.16)  0.14 (0.01, 2.60) | 0.48  0.19 | 0% |
| Agent (Pneumonia)  Metoclopramide  Erythromycin | 525  68 | 1.08 (0.78, 1.49)  0.81 (0.48, 1.38) | 0.64  0.44 | 0% |
| Agent (Feeding Intolerance)  Metoclopramide  Erythromycin | 19  40 | 0.45 (0.05, 4.16)  0.14 (0.01, 2.60) | 0.48  0.19 | 0% |

GRV: gastric residual volume

In this table we present the estimates for all subgroup analyses, we present the estimates for each subgroup with p-values, in addition we present the I^2^ value for between groups heterogeneity (larger values correlate with greater heterogeneity). The analyses are limited by small number of trials, therefore, we can not definitely rule out significant subgroup differences.

**Table 4.** **Risk of Bias**

| **Study** | **Random sequence generation** | **Allocation concealment** | **Blinding of participants and personal (performance bias)** | **Blinding of outcome data** | **Incomplete outcome data** | **Selective reporting bias** | **Other** | **Overall risk** |
| --- | --- | --- | --- | --- | --- | --- | --- | --- |
| **Whatley 1984** | Low | Low | Unclear | Unclear | Low | Low | Low | Unclear |
| **Heiselman 1995** | Unclear | Unclear | Unclear | Unclear | Low risk | Low | Low | Unclear |
| **Kalliafas 1996** | Low | Low | Low | Low | Low | Low | Low | Low |
| **Paz 1996** | Low | Low | Low | Low | Low | Low | Low | Low |
| **Chapman 2000** | Unclear | Unclear | Low | Unclear | Low | Low | Low | Unclear |
| **Yavagal 2000** | Unclear | Unclear | Unclear | Unclear | Low | Low | Low | Unclear |
| **Pinilla 2001** | Low | Low | Low | Low | Low | Low | Low | Low |
| **Berne 2002** | Unclear | Unclear | Low | Low | Low | Low | Low | Unclear |
| **Reignier 2002** | Unclear | Low | High | High | Low | Low | Low | High |
| **Griffith 2003** | Unclear | Unclear | Low | Low | Low | Low | Low | Unclear |
| **Nursal 2007** | Low | Low | Low | Low | Low | Low | Low | Low |
| **Nassaj 2010** | High | High | Low | Unclear | Low | Low | Low | High |
| **Hu 2015** | Low | Low | Unclear | Low | Low | Low | Low | Low |

**Table 5. Quality of Evidence**

| **Quality assessment** | | | | | | | **№ of patients** | | **Effect** | | **Quality** | **Importance** |
| --- | --- | --- | --- | --- | --- | --- | --- | --- | --- | --- | --- | --- |
| **№ of studies** | **Study design** | **Risk of bias** | **Inconsistency** | **Indirectness** | **Imprecision** | **Other considerations** | **prokinetic agents** | **placebo** | **Relative (95% CI)** | **Absolute (95% CI)** |  |  |
| **Pneumonia** | | | | | | | | | | | | |
| 3 | randomised trials | serious ^1^ | not serious | not serious ^2^ | serious ^3^ | none ^4^ | 58/231 (25.1%) | 93/362 (25.7%) | **RR 1.00** (0.76 to 1.32) | **0 fewer per 1,000** (from 62 fewer to 82 more) | ⨁⨁◯◯ LOW | CRITICAL |
| **Mortality** | | | | | | | | | | | | |
| 6 | randomised trials | not serious | not serious | not serious ^2^ | serious ^5^ | none ^4^ | 105/271 (38.7%) | 166/401 (41.4%) | **RR 0.97** (0.81 to 1.16) | **12 fewer per 1,000** (from 66 more to 79 fewer) | ⨁⨁⨁◯ MODERATE | CRITICAL |
| **Feeding Intolerance** | | | | | | | | | | | | |
| 6 | randomised trials | not serious | not serious | not serious | serious ^6^ | none ^4^ | 47/116 (40.5%) | 61/111 (55.0%) | **RR 0.73** (0.55 to 0.97) | **148 fewer per 1,000** (from 16 fewer to 247 fewer) | ⨁⨁⨁◯ MODERATE | CRITICAL |
| **High Gastric Residual Volumes** | | | | | | | | | | | | |
| 5 | randomised trials | not serious | not serious | not serious | serious ^7^ | none ^4^ | 44/116 (37.9%) | 62/111 (55.9%) | **RR 0.69** (0.52 to 0.91) | **173 fewer per 1,000** (from 50 fewer to 268 fewer) | ⨁⨁⨁◯ MODERATE | CRITICAL |
| **Success of Post-Pyloric Feeding Tube Placement** | | | | | | | | | | | | |
| 6 | randomised trials | serious ^8^ | not serious ^9^ | not serious | not serious | none ^4^ | 174/349 (49.9%) | 71/214 (33.2%) | **RR 1.60** (1.17 to 2.21) | **199 more per 1,000** (from 56 more to 401 more) | ⨁⨁⨁◯ MODERATE | IMPORTANT |
| **ICU Length of Stay** | | | | | | | | | | | | |
| 2 | randomised trials | not serious | not serious | not serious | very serious ^10^ | none ^4^ | 42 | 45 | - | MD **1.24 more** (5.21 fewer to 7.68 more) | ⨁⨁◯◯ LOW | IMPORTANT |

**CI:** Confidence interval; **RR:** Risk ratio; **MD:** Mean difference

1. We downgraded the quality of evidence by one level for risk of bias, the included were not properly blinded
2. Although the studies included any critically ill patient, we did not downgrade for indirectness
3. We downgraded for imprecision by one level, the CI included both significant benefit and harm
4. We did not downgrade for publication bias, although we could not assess this category reliably due to small number of eligible studies
5. We downgraded the quality of evidence by one level for imprecision, the CI contained significant harm
6. We downgraded the quality of evidence by one level for imprecision, the CI contained small benefit that did not meet the clinical decision threshold
7. We downgraded the quality of evidence by one level for imprecision, the CI contained small benefit that did not meet the clinical decision threshold
8. We downgraded the quality of evidence by one level for risk of bias, blinding of outcome assessors and healthcare workers was not appropriate in majority of studies
9. I^2^=45% and Chi^2^ = 0.11, we did not downgrade for inconsistency
10. We downgraded the quality of evidence by two levels for imprecision, the CI is very wide containing extreme benefit and harm

**Table 6. PRISMA Checklist**

| **Section/topic** | **#** | **Checklist item** | **Reported on page #** |
| --- | --- | --- | --- |
| **TITLE** | | |  |
| Title | 1 | Identify the report as a systematic review, meta-analysis, or both. | 1 |
| **ABSTRACT** | | |  |
| Structured summary | 2 | Provide a structured summary including, as applicable: background; objectives; data sources; study eligibility criteria, participants, and interventions; study appraisal and synthesis methods; results; limitations; conclusions and implications of key findings; systematic review registration number. | 3  Systematic review registration number N/A |
| **INTRODUCTION** | | |  |
| Rationale | 3 | Describe the rationale for the review in the context of what is already known. | 4-5 |
| Objectives | 4 | Provide an explicit statement of questions being addressed with reference to participants, interventions, comparisons, outcomes, and study design (PICOS). | 5 |
| **METHODS** | | |  |
| Protocol and registration | 5 | Indicate if a review protocol exists, if and where it can be accessed (e.g., Web address), and, if available, provide registration information including registration number. | N/A |
| Eligibility criteria | 6 | Specify study characteristics (e.g., PICOS, length of follow-up) and report characteristics (e.g., years considered, language, publication status) used as criteria for eligibility, giving rationale. | 6 |
| Information sources | 7 | Describe all information sources (e.g., databases with dates of coverage, contact with study authors to identify additional studies) in the search and date last searched. | 6 |
| Search | 8 | Present full electronic search strategy for at least one database, including any limits used, such that it could be repeated. | Appendix Table 1 |
| Study selection | 9 | State the process for selecting studies (i.e., screening, eligibility, included in systematic review, and, if applicable, included in the meta-analysis). | 6 |
| Data collection process | 10 | Describe method of data extraction from reports (e.g., piloted forms, independently, in duplicate) and any processes for obtaining and confirming data from investigators. | 6 |
| Data items | 11 | List and define all variables for which data were sought (e.g., PICOS, funding sources) and any assumptions and simplifications made. | 6, 14 |
| Risk of bias in individual studies | 12 | Describe methods used for assessing risk of bias of individual studies (including specification of whether this was done at the study or outcome level), and how this information is to be used in any data synthesis. | 7 |
| Summary measures | 13 | State the principal summary measures (e.g., risk ratio, difference in means). | 7 |
| Synthesis of results | 14 | Describe the methods of handling data and combining results of studies, if done, including measures of consistency (e.g., I^2^) for each meta-analysis. | 7 |

| **Section/topic** | **#** | **Checklist item** | **Reported on page #** |
| --- | --- | --- | --- |
| Risk of bias across studies | 15 | Specify any assessment of risk of bias that may affect the cumulative evidence (e.g., publication bias, selective reporting within studies). | 7 |
| Additional analyses | 16 | Describe methods of additional analyses (e.g., sensitivity or subgroup analyses, meta-regression), if done, indicating which were pre-specified. | 7-8 |
| **RESULTS** | | |  |
| Study selection | 17 | Give numbers of studies screened, assessed for eligibility, and included in the review, with reasons for exclusions at each stage, ideally with a flow diagram. | 8 |
| Study characteristics | 18 | For each study, present characteristics for which data were extracted (e.g., study size, PICOS, follow-up period) and provide the citations. | Table 1 |
| Risk of bias within studies | 19 | Present data on risk of bias of each study and, if available, any outcome level assessment (see item 12). | Figure 2, Appendix Table 4 and 5 |
| Results of individual studies | 20 | For all outcomes considered (benefits or harms), present, for each study: (a) simple summary data for each intervention group (b) effect estimates and confidence intervals, ideally with a forest plot. | 9-10 |
| Synthesis of results | 21 | Present results of each meta-analysis done, including confidence intervals and measures of consistency. | 9-10 |
| Risk of bias across studies | 22 | Present results of any assessment of risk of bias across studies (see Item 15). | 10 |
| Additional analysis | 23 | Give results of additional analyses, if done (e.g., sensitivity or subgroup analyses, meta-regression [see Item 16]). | 10 |
| **DISCUSSION** | | |  |
| Summary of evidence | 24 | Summarize the main findings including the strength of evidence for each main outcome; consider their relevance to key groups (e.g., healthcare providers, users, and policy makers). | 10 |
| Limitations | 25 | Discuss limitations at study and outcome level (e.g., risk of bias), and at review-level (e.g., incomplete retrieval of identified research, reporting bias). | 13 |
| Conclusions | 26 | Provide a general interpretation of the results in the context of other evidence, and implications for future research. | 13 |
| **FUNDING** | | |  |
| Funding | 27 | Describe sources of funding for the systematic review and other support (e.g., supply of data); role of funders for the systematic review. | 14 |

*From:*  Moher D, Liberati A, Tetzlaff J, Altman DG, The PRISMA Group (2009). Preferred Reporting Items for Systematic Reviews and Meta-Analyses: The PRISMA Statement. PLoS Med 6(7): e1000097. doi:10.1371/journal.pmed1000097

**Figure 1.** **Funnel Plot (Feeding Intolerance Outcome)**


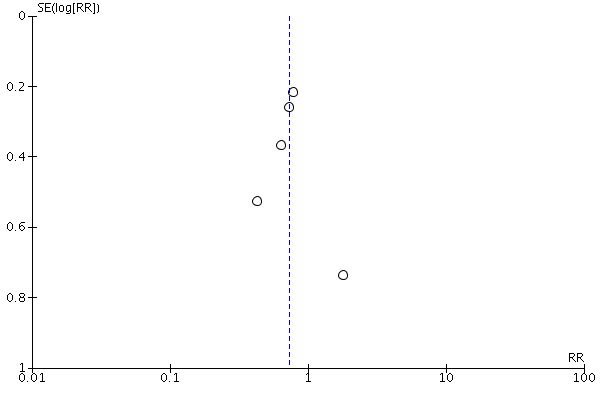


SE: standard error; RR: relative risk

Funnel plot that present individual estimates of studies, Y-axis SE (log[RR]) values are inversely proportional to study sample size; X-axis represent the point estimate for individual studies. The number of studies is small, therefore, this too is underpowered to properly investigate for presence of publication bias.

**Figure 2. Funnel Plot (High GRV)**

**
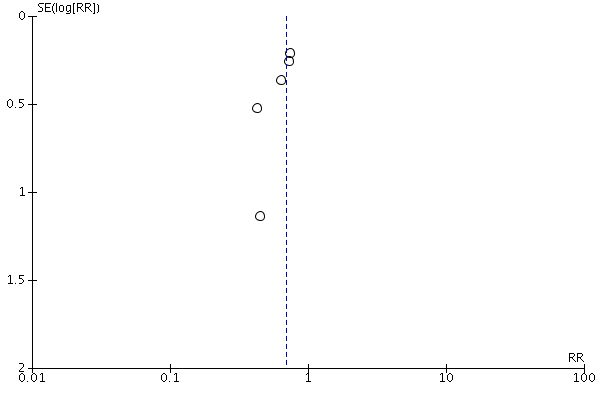
**

SE: standard error; RR: relative risk

Funnel plot that present individual estimates of studies, Y-axis SE (log[RR]) values are inversely proportional to study sample size; X-axis represent the point estimate for individual studies. The number of studies is small, therefore, this too is underpowered to properly investigate for presence of publication bias.

**Figure 3. Mortality Outcome**

**
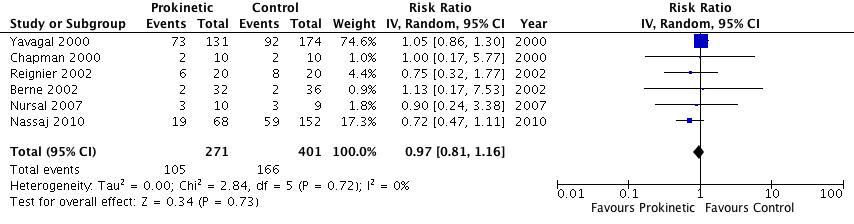
**Legend:

IV: inverse variance; CI: confidence interval

**Figure 4. ICU Length of Stay Outcome**

**
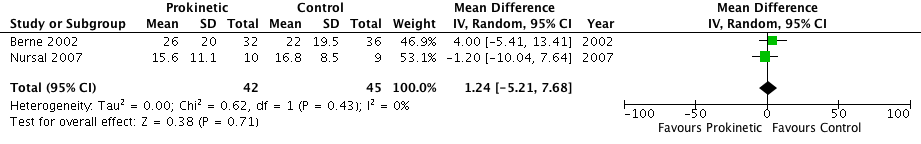
**

Legend:

IV: inverse variance; CI: confidence interval

**Figure 5. Vomiting Outcome**


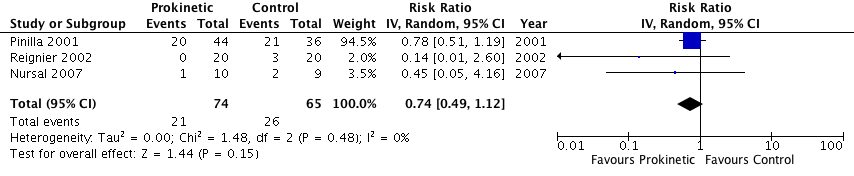


Legend:

IV: inverse variance; CI: confidence interval

**Figure 6.** **Diarrhea Outcome**


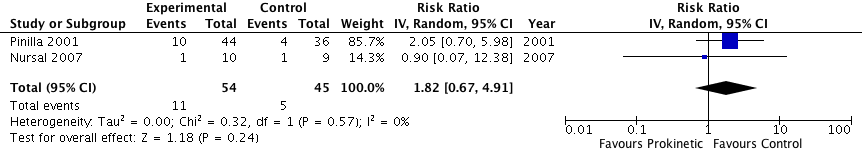


Legend:

IV: inverse variance; CI: confidence interval

**Figure 7.** **Subgroup Analysis by GRV Threshold**


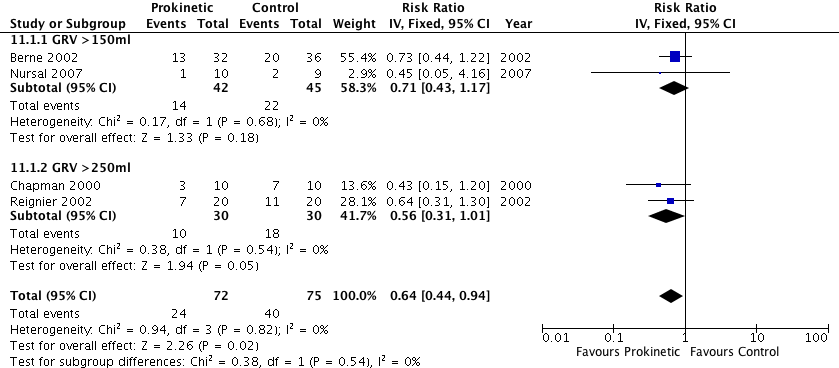


This Forrest plot demonstrate the results of subgroup analysis by gastric residual volume (defined as greater than 150cc vs 250cc), the interaction p value and I^2^ for between groups heterogeneity were not significant.

**Figure 8.** **Subgroup Analysis by Indication of Treatment**


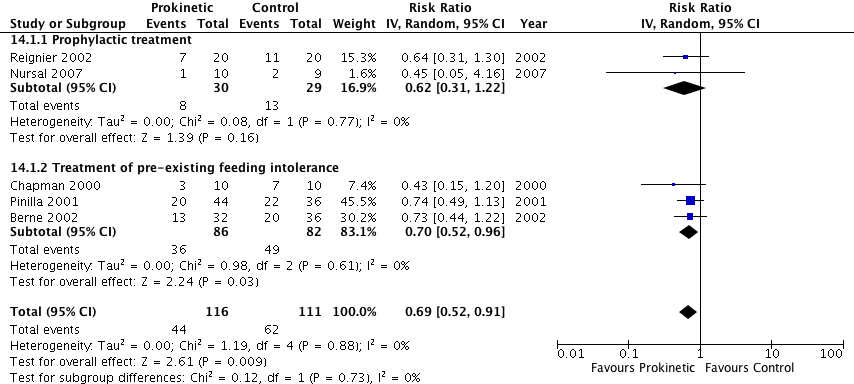


IV: inverse variance; CI: confidence interval

This Forrest plot demonstrate the results of subgroup analysis by indication of treatment (prophylaxis vs. treatment), the interaction p value and I^2^ for between groups heterogeneity were not significant.

**Figure 9. Subgroup Analyses by Risk of Bias (Feeding Intolerance Outcome)**


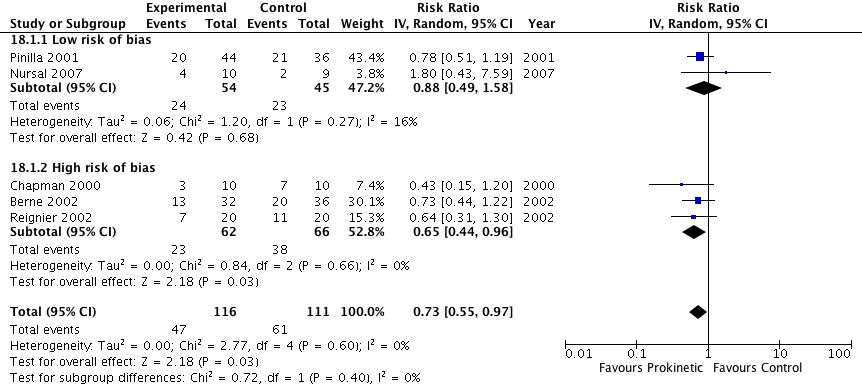


This Forrest plot demonstrate the results of subgroup analysis by risk of bias (low risk vs. high or unclear risk), the interaction p value and I^2^ for between groups heterogeneity were not significant.

IV: inverse variance; CI: confidence interval

**Figure10. Subgroup Analysis by Drug Class**

**A) Mortality Outcome**


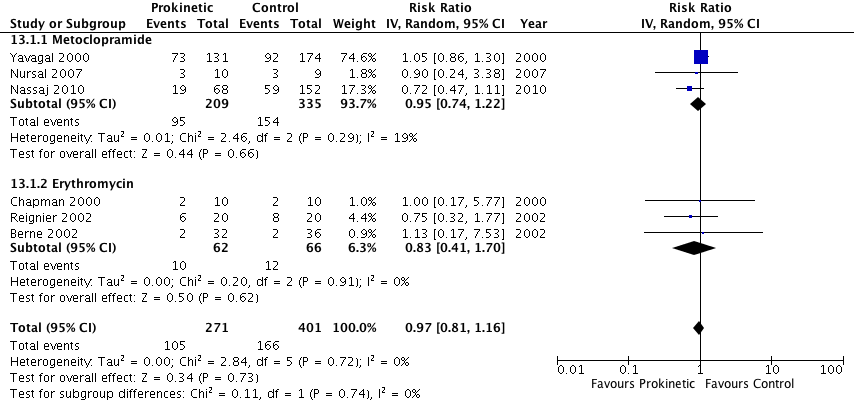


IV: inverse variance; CI: confidence interval

This Forrest plot demonstrate the results of subgroup analysis by drug class for mortality (first subgroup metoclopramide vs. placebo or no treatment; second subgroup erythromycin vs. placebo or no treatment), the interaction p value and I^2^ for between groups heterogeneity were not significant.

**B) Feeding Intolerance Outcome**


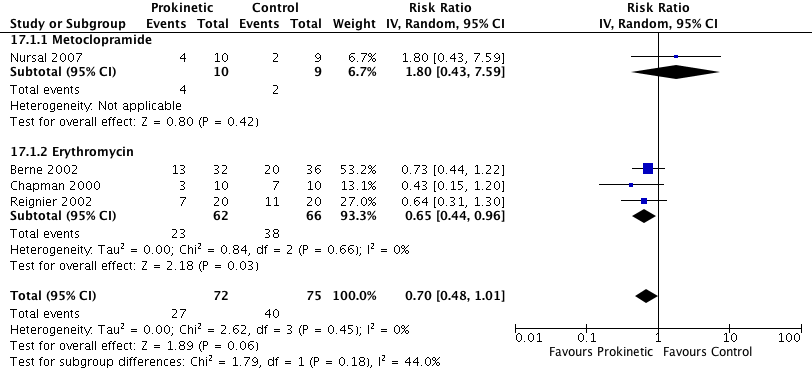


IV: inverse variance; CI: confidence interval

This Forrest plot demonstrate the results of subgroup analysis by drug class for feeding intolerance (first subgroup metoclopramide vs. placebo or no treatment; second subgroup erythromycin vs. placebo or no treatment), the interaction p value and I^2^ for between groups heterogeneity were not significant.
